# Transcriptome Analysis Reveals Candidate Pathways and Genes Involved in Wheat (*Triticum aestivum* L.) Response to Zinc Deficiency

**DOI:** 10.3390/biology14080985

**Published:** 2025-08-02

**Authors:** Shoujing Zhu, Shiqi Zhang, Wen Wang, Nengbing Hu, Wenjuan Shi

**Affiliations:** College of Agriculture, Anhui Science and Technology University, Chuzhou 233100, China; zhusj@ahstu.edu.cn (S.Z.); zhangshiqi_wheat@sina.com (S.Z.); wangwen291@sina.com (W.W.); hunb@ahstu.edu.cn (N.H.)

**Keywords:** *Triticum aestivum* L., zinc deficiency, transcriptome, zinc ion transport, phenylpropanoid biosynthesis pathway, nicotianamine, ethylene biosynthesis

## Abstract

Zinc (Zn) deficiency poses a major global health challenge, and wheat grains generally contain low Zn concentrations. In this study, the wheat cultivar ‘Zhongmai 175’ was identified as zinc-efficient. Hydroponic experiments demonstrated that Zn deficiency induced the secretion of oxalic acid and malic acid in root exudates and significantly increased total root length in ‘Zhongmai 175’. To elucidate the underlying regulatory mechanisms, transcriptome profiling via RNA sequencing was conducted under Zn-deficient conditions. A total of 2287 and 1935 differentially expressed genes (DEGs) were identified in roots and shoots, respectively. Gene Ontology enrichment analysis revealed that these DEGs were primarily associated with Zn ion transport, homeostasis, transmembrane transport, and hormone signaling. Key DEGs belonged to gene families including *VIT*, *NAS*, *DMAS*, *ZIP*, *tDT*, *HMA*, and *NAAT*. KEGG pathway analysis indicated that phenylpropanoid biosynthesis, particularly lignin synthesis genes, was significantly downregulated in Zn-deficient roots. In shoots, cysteine and methionine metabolism, along with plant hormone signal transduction, were the most enriched pathways. Notably, most DEGs in shoots were associated with the biosynthesis of phytosiderophores (MAs, NA) and ethylene. Overall, genes involved in Zn ion transport, phytosiderophore biosynthesis, dicarboxylate transport, and ethylene biosynthesis appear to play central roles in wheat’s adaptive response to Zn deficiency. These findings provide a valuable foundation for understanding the molecular basis of Zn efficiency in wheat and for breeding Zn-enriched varieties.

## Simple Summary

Zinc deficiency is a serious global health problem, partly because staple foods like wheat often lack sufficient zinc. This study investigated how a specific wheat variety (‘Zhongmai 175’) copes well with low zinc levels. Experiments showed that when zinc was scarce, these wheat plants released organic acids (oxalic and malic acid) from their roots and grew longer roots. To understand why, we studied how zinc deficiency changed the activity of genes in the plant’s roots and aboveground parts. Many genes showed different activity levels. Key genes involved were those responsible for moving zinc within the plant, maintaining zinc balance, transporting molecules across cell membranes, and responding to plant hormones. In roots, genes linked to lignin biosynthesis were less active. In aboveground parts, genes involved in phytosiderophores and ethylene synthesis were more active. Overall, zinc movement, production of these grabbing molecules, acid release, and ethylene hormone signals are crucial for wheat’s zinc efficiency. Understanding these mechanisms helps scientists breed new wheat varieties with adequate zinc content in their grains, which can improve nutrition and combat zinc deficiency worldwide.

## 1. Introduction

Zinc (Zn) is an essential micronutrient required for numerous biological processes in both plants and animals. Globally, Zn deficiency affects approximately 2 billion people, contributing significantly to disease and mortality rates [1]. The primary causes are monotonous diets and insufficient dietary Zn intake [2]. Wheat (*Triticum aestivum* L.), one of the three major staple cereals, accounts for 37.5% of global cereal cultivation area and contributes 28.7% to total cereal production (FAOSTAT 2019). It supplies 20% of the world’s dietary energy and protein, and roughly 40% of Zn intake [3]. However, since the Green Revolution, increased wheat yields have been accompanied by a marked decline in grain Zn concentration—a phenomenon known as the “yield dilution effect” [4,5]. The global average Zn content in wheat grains ranges from 20 to 30 mg·kg^−1^, and in Zn-deficient soils, it can drop below 10 mg·kg^−1^ [6,7], far below the recommended dietary level of 40–60 mg·kg^−1^ [8]. In China, wheat grain Zn concentrations vary from 13 to 85 mg·kg^−1^, averaging 31 mg·kg^−1^, with only 4% of samples exceeding the recommended threshold of 40 mg·kg^−1^ [9]. Therefore, enhancing Zn accumulation in wheat grains is critical for combating global Zn deficiency.

Zinc in wheat grains is derived from two primary sources: direct root uptake and remobilization from vegetative tissues during grain filling. Under controlled conditions, the relative contribution of these sources is influenced by Zn and nitrogen nutritional status [10]. In field conditions, remobilization generally accounts for over 60% of grain Zn content [11]. The movement of Zn from the soil to grains involves a complex array of physiological and biochemical processes, including transmembrane transport, long-distance movement, loading/unloading, intracellular homeostasis, and Zn ligand metabolism [12,13]. Zn (II) uptake occurs through root plasma membranes via ZIP family transporters (ZRT, IRT-like proteins) or as chelates with phytosiderophores such as deoxymugineic acid (DMA), which are transported by Yellow Stripe-Like (YSL) proteins [14,15]. Some Zn is sequestered in root vacuoles via metal tolerance proteins (MTPs) or heavy metal ATPases (HMAs) [16], while another portion forms complexes with nicotianamine (NA) and moves symplastically through plasmodesmata to the root pericycle.

For root-to-shoot transport, Zn is loaded into the xylem via HMA-mediated transmembrane transport. Within the xylem, Zn forms complexes with citrate and moves with the transpiration stream to aerial tissues. Zn is then unloaded into these organs across membranes. At vascular bundle interfaces between xylem and phloem, lateral transport occurs: Zn (II) is transferred to the phloem via ZIP transporters. In the phloem, Zn binds NA or peptides for transport to the grain, where it is ultimately unloaded [14]. During grain filling, Zn stored in senescing vegetative tissues is remobilized to developing grains via the phloem. Zn is loaded into the phloem from mesophyll, sheath cells, cytosol, and vacuoles, facilitated by ZIP transporters, and then chelated with NA or peptides for delivery to grains [14]. Within the grain, Zn distribution is governed by additional mechanisms. In the outer aleurone layers, phytic acid and protein storage vacuoles (PSVs) regulate Zn (II) levels via MTPs, contributing to lower Zn concentration in the starchy endosperm [14,17]. Genetic variation among wheat cultivars in any of these steps can result in substantial differences in grain Zn accumulation.

Thus, a comprehensive understanding of the physiological and molecular mechanisms underlying Zn uptake, translocation, remobilization, and storage is crucial for deciphering genotypic variation and breeding Zn-enriched wheat. Despite considerable progress, key regulatory mechanisms governing Zn homeostasis in wheat remain poorly defined. RNA sequencing (RNA-seq) offers a powerful approach to identify genes and pathways involved in stress responses and has been widely employed to study wheat under various abiotic stresses [18,19,20,21]. In this study, we assessed the grain Zn content of 42 widely cultivated wheat cultivars in China and selected the high-efficiency cultivar ‘Zhongmai 175’ for transcriptomic analysis under Zn-deficient conditions. GO and KEGG analyses revealed that genes related to organic acid metabolism, tonoplast dicarboxylate transporters, metal ion transporters, phytosiderophores, and hormone signaling play pivotal roles in the wheat response to Zn deficiency. These findings offer valuable insights for future genetic improvement and breeding of Zn-efficient wheat varieties.

## 2. Results

### 2.1. Zinc Concentration in Wheat Grain

**Figure 1:**
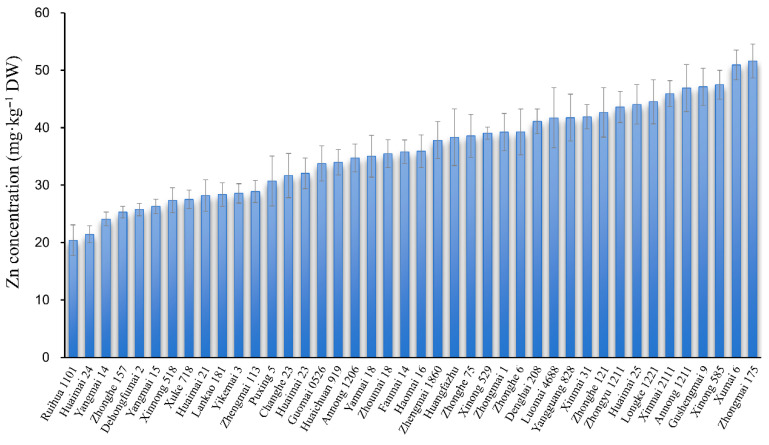
Grain zinc concentrations of 42 wheat cultivars.

The zinc concentration of grains from 42 Chinese common wheat cultivars grown under uniform field conditions was measured (Figure 1). Grain Zn levels ranged from 20.41 to 51.59 mg·kg^−1^, with an average of 36.06 mg·kg^−1^. Fourteen cultivars met or exceeded the recommended threshold of 40 mg·kg^−1^, with Zhongmai 175 and Xumai 16 exhibiting the highest Zn contents at 51.59 and 50.94 mg·kg^−1^, respectively. In contrast, 28 cultivars fell below the recommended value.

**Figure 2:**
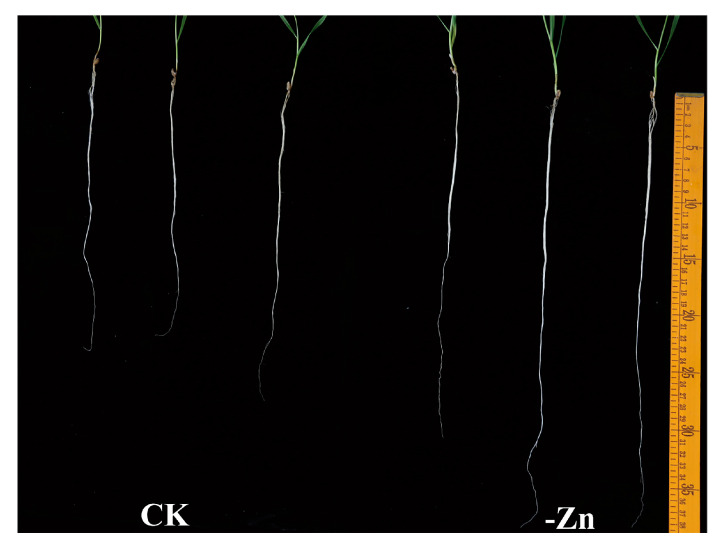
Root morphology of Zhongmai 175 under Zn-sufficient (CK, 2 µM) and Zn-deficient (-Zn, 0 µM) conditions.

**Figure 3:**
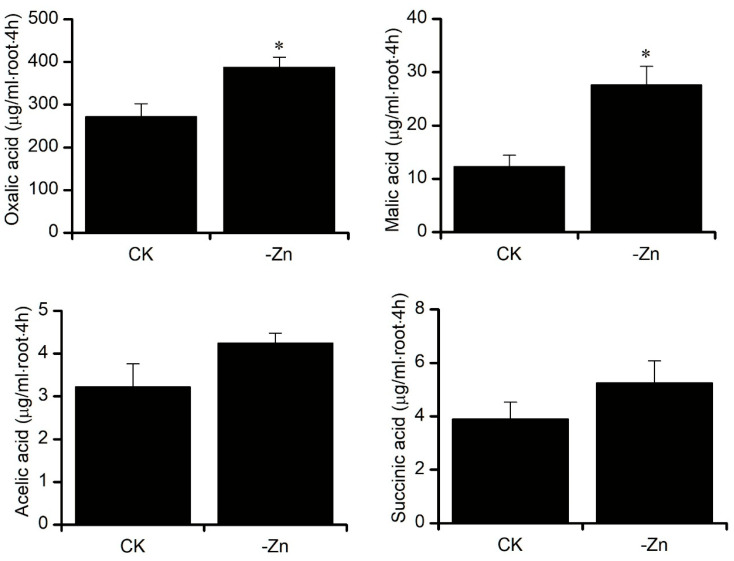
Organic acid content in root exudates of Zhongmai 175 under Zn-sufficient (CK) and Zn-deficient (-Zn) conditions. Different letters indicate significant differences (*p* < 0.05). Asterisks (*) denote significant changes compared to CK (*p* < 0.05). Data are means of three replicates.

To investigate the response of Zhongmai 175 to Zn deficiency, hydroponic experiments were conducted. Compared with Zn-sufficient conditions (CK), total root length increased significantly under Zn-deficient treatment (-Zn) (Figure 2), while total root surface area, root volume, average diameter, and number of root tips showed no significant differences (Table 1). Further analysis revealed that Zn deficiency induced the secretion of four types of organic acids in root exudates, with oxalic acid and malic acid significantly elevated by 1.43-fold and 2.25-fold, respectively (Figure 3).

### 2.2. RNA-Seq Data Quality Assessment

**Figure 4:**
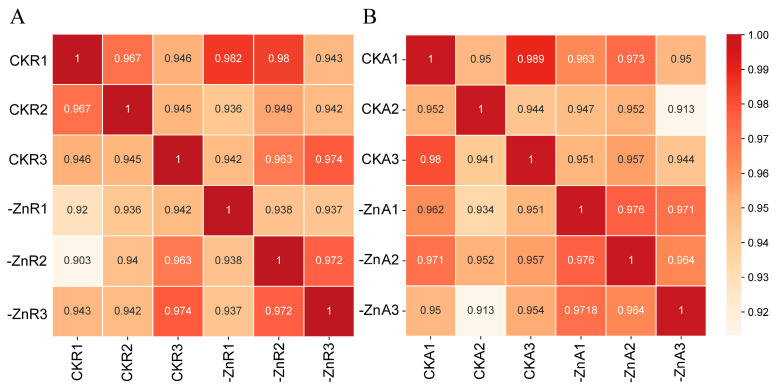
Pearson correlation heatmaps among biological replicates. (**A**) Root samples (CKR vs. -ZnR). (**B**) Aboveground samples (CKA vs. -ZnA). CKR: roots under Zn-sufficient conditions; -ZnR: roots under Zn-deficient conditions; CKA: aboveground parts under Zn-sufficient conditions; -ZnA: aboveground parts under Zn-deficient conditions.

To uncover molecular mechanisms underlying Zn uptake and accumulation in Zhongmai 175, transcriptome sequencing was performed on roots and aboveground tissues collected at the two-leaf stage under both Zn-sufficient and Zn-deficient conditions. RNA-seq libraries were constructed from 12 samples, and sequencing data quality was assessed (Table 2). Clean reads aligned to 85.39–97.70% of the *Triticum aestivum* reference genome, with approximately 82% mapping to exon regions. Pearson correlation coefficients among replicates ranged from 0.913 to 0.989, indicating high reproducibility (Figure 4). These data support the reliability of the transcriptome results for subsequent analyses. All raw sequencing data are available in the NCBI Sequence Read Archive under accession number PRJNA1290848.

### 2.3. Differentially Expressed Gene (DEG) Analysis

Gene expression levels were quantified using fragments per kilobase of transcript per million mapped reads (FPKM). Genes with FPKM ≥ 1 in at least two samples were considered expressed. Differentially expressed genes (DEGs) were defined as those with |log_2_ (fold change) | > 1 and *p* < 0.05.

**Figure 5:**
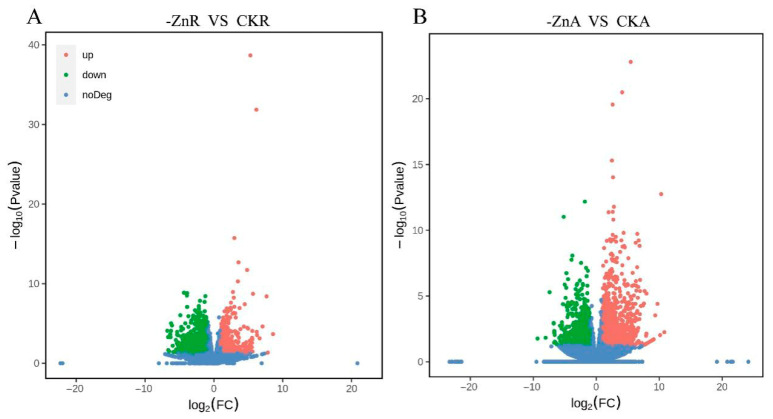
Volcano plots of DEGs under Zn deficiency. (**A**) Roots: zinc deficiency (-ZnR) versus those grown under normal conditions (CKR). (**B**) Aboveground: zinc deficiency (-ZnA) versus those grown under normal conditions (CKA).

In total, 2287 DEGs were identified in roots between Zn-deficient and control conditions, with 1375 upregulated and 912 downregulated (Figure 5A; Table S2). In aboveground tissues, 1935 DEGs were detected, including 734 upregulated and 1202 downregulated genes under Zn deficiency (Figure 5B; Table S3).

### 2.4. GO Enrichment of DEGs

To explore the biological functions of differentially expressed genes (DEGs) under zinc deficiency, Gene Ontology (GO) enrichment analysis was conducted for both root and aboveground tissues. In roots, DEGs were significantly enriched in 14 biological processes, nine cellular components, and nine molecular functions. The most overrepresented biological processes included ion transmembrane transport (GO: 0034220), response to metal ion (GO: 0010038), metal ion transport (GO: 0030001), inorganic ion homeostasis (GO: 0098771), zinc ion transport (GO: 0006829), and zinc ion homeostasis (GO: 0055069). Additional enriched terms included cellular response to hormone stimulus (GO: 0032870), response to acidic chemical (GO: 0071229), secondary metabolic process (GO: 0019748), and carboxylic acid biosynthetic process (GO: 0046394).

**Figure 6:**
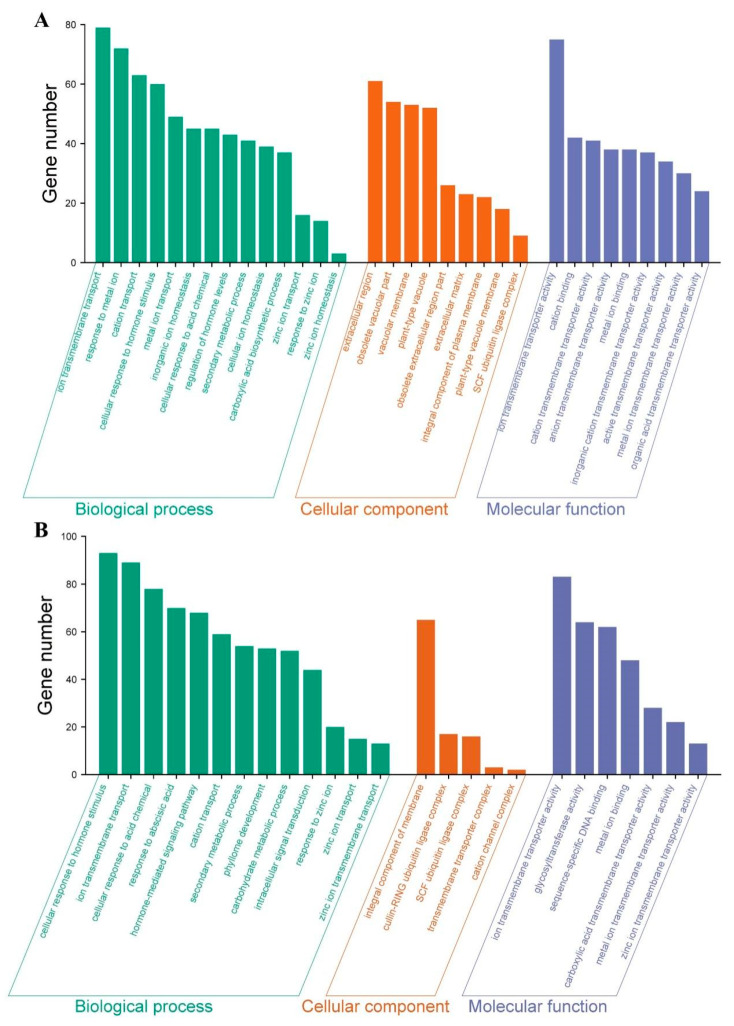
GO classification of DEGs. (**A**) DEGs from root tissues (-ZnR vs. CKR). (**B**) DEGs from aboveground tissues (-ZnA vs. CKA). CKR: roots under Zn-sufficient conditions; -ZnR: roots under Zn-deficient conditions; CKA: aboveground parts under Zn-sufficient conditions; -ZnA: aboveground parts under Zn-deficient conditions.

For cellular components, DEGs were significantly associated with the extracellular region, vacuolar membrane, plant-type vacuole, extracellular matrix, integral component of the plasma membrane, and plant-type vacuole membrane. In terms of molecular function, DEGs were predominantly enriched in metal ion binding (GO: 0046872) and ion transmembrane transporter activity (GO: 0015075) (Figure 6A).

In aboveground tissues, DEGs were annotated to 13 biological processes, five cellular components, and seven molecular functions. Key biological processes included response to hormone stimulus (GO: 0032870), ion transmembrane transport (GO: 0008509), response to acidic chemical (GO: 0071229), response to abscisic acid (GO: 0009737), hormone-mediated signaling pathway (GO: 0009755), cation transport (GO: 0006812), secondary metabolism (GO: 0019748), phyllome development (GO: 0048827), response to zinc ion (GO: 0010043), and zinc ion transport (GO: 0006829). DEGs were also enriched in cellular components such as membranes, ubiquitin ligase complexes, transmembrane transporter complexes, and cation channel complexes. Molecular function terms included metal ion binding (GO: 0046872), zinc ion transmembrane transporter activity (GO: 0005385), DNA binding (GO: 0043565), and glycosyltransferase activity (GO: 0016757) (Figure 6B).

### 2.5. Analysis of DEGs Related to Zinc Ion Transport

Zinc transport from roots to shoots, as well as its redistribution via the phloem, occurs through Zn^2+^ ions or Zn-organic complexes [3]. These processes rely heavily on transporter families such as ZIP (ZRT-IRT-like proteins) [15], heavy metal ATPases (HMA) [22,23], and organic acid chelators like mugineic acid (MAs) [24], citric acid [25], and malic acid [26,27].

In the root transcriptome, Zn deficiency significantly induced 2 DEGs encoding vacuolar iron transporters (VIT), 4 DEGs encoding nicotianamine synthase (NAS), 1 DEG encoding deoxymugineic acid synthase (DMAS), 5 DEGs encoding ZIP transporters, 4 DEGs encoding tonoplast dicarboxylate transporters (tDT), and 1 DEG encoding cadmium/zinc-transporting ATPase 2 (HMA2).

In aboveground tissues, five ZIP-encoding DEGs, five tDT-encoding DEGs, 2 HMAs, one basic leucine zipper transcription factor (bZIP19), and 1 DEG encoding nicotianamine aminotransferase A (NAAT) were significantly upregulated under Zn deficiency. Among these, *VIT1* (*TraesCS7D02G413000*) and *tDT* (*TraesCS3D02G188000*) showed particularly high fold changes (6.17 and 10.32, respectively) with significant *P*-values (1.39e^−32^ and 1.78e^−13^). These results suggest that metal ion transporters (ZIP, VIT, ABC, and HMA) and genes involved in organic acid synthesis and transport (NAS, NAAT, and tDT) are crucial for Zn uptake and redistribution under Zn-deficient conditions (Table 3).

### 2.6. KEGG Pathway Enrichment of DEGs

**Figure 7:**
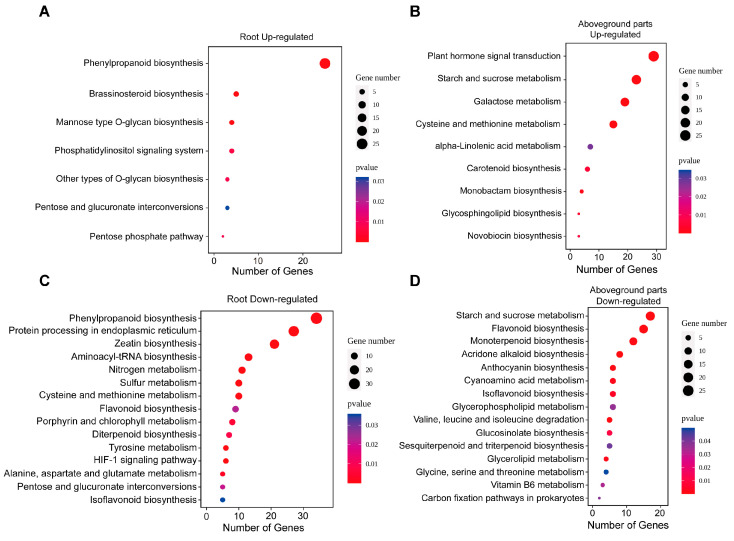
KEGG pathway enrichment of DEGs under Zn deficiency. (**A**) Upregulated DEGs in roots. (**B**) Upregulated DEGs in aboveground tissues. (**C**) Downregulated DEGs in roots. (**D**) Downregulated DEGs in aboveground tissues.

To identify the biological pathways involved in wheat’s response to Zn deficiency, KEGG enrichment analysis was conducted using KOBAS v3.0. In roots, downregulated DEGs were enriched in a broader array of pathways compared to upregulated DEGs, with 7 and 15 enriched pathways, respectively (Figure 7A,C). Upregulated DEGs were mainly enriched in the “phenylpropanoid biosynthesis” pathway, which included genes involved in lignin biosynthesis such as *Cinnamoyl-CoA reductase* (CCR), *Cinnamyl alcohol dehydrogenase* (CAD), *Caffeic acid O-methyltransferase* (COMT), *Peroxidases* (POD), and *Laccases* (LAC).

Conversely, downregulated DEGs in roots were enriched in pathways related to mRNA translation and protein processing, including “protein processing in the endoplasmic reticulum” and “aminoacyl-tRNA biosynthesis”. Additional downregulated pathways included “zeatin biosynthesis”, “nitrogen metabolism”, “sulfur metabolism”, and “cysteine and methionine metabolism” (Tables S3 and S6).

In the aboveground parts, nine upregulated and 15 downregulated KEGG pathways were identified (Figure 7B,D). Upregulated DEGs were mainly enriched in “plant hormone signal transduction”, “starch and sucrose metabolism”, “galactose metabolism”, and “cysteine and methionine metabolism”. In the hormone signaling pathway, genes encoding auxin-responsive proteins (*IAA*, *SAUR*), ethylene-insensitive protein 3 (*EIN3*), and ethylene receptor (*ETR*) were upregulated. In the cysteine and methionine pathway, DEGs included *1-aminocyclopropane-1-carboxylate oxidase* (*ACO*), *1-aminocyclopropane-1-carboxylate synthase* (*ACS*), and *S-adenosylmethionine synthase* (*SAMS*).

Downregulated DEGs in aboveground tissues were primarily enriched in secondary metabolic processes such as “flavonoid biosynthesis”, “monoterpenoid biosynthesis”, “acridone alkaloid biosynthesis”, “anthocyanin biosynthesis”, and “isoflavonoid biosynthesis” (Tables S3 and S7).

### 2.7. Downregulation of Lignin Biosynthesis-Related DEGs Under Zinc Deficiency

KEGG pathway analysis revealed that the phenylpropanoid biosynthesis pathway was the most significantly enriched in wheat roots under zinc-deficient conditions. This pathway is central to the production of a wide array of secondary metabolites derived from phenylalanine and tyrosine, including lignin and flavonoids, as well as several intermediates with largely unknown biological roles. Lignin is categorized into three types based on its monomer composition: p-hydroxyphenyl lignin (H-lignin), guaiacyl lignin (G-lignin), and syringyl lignin (S-lignin). The enzymes cinnamoyl-CoA reductase (CCR), cinnamyl alcohol dehydrogenase (CAD), and caffeic acid O-methyltransferase (COMT) are key to the biosynthesis of these lignin monomers.

**Figure 8:**
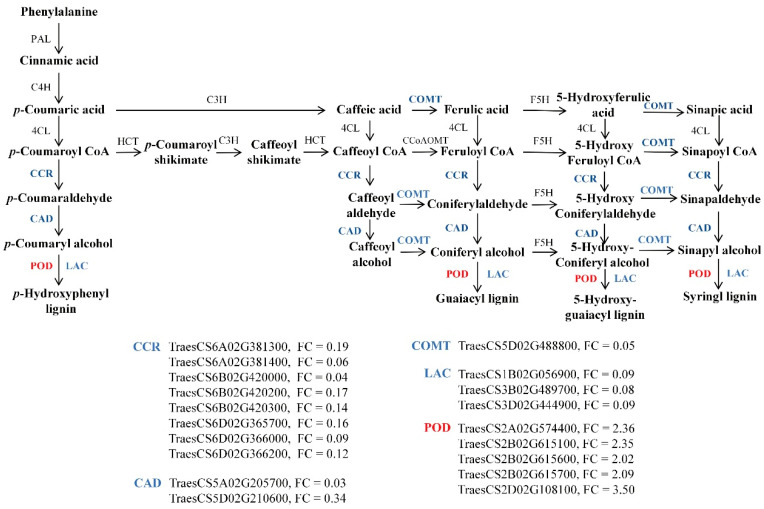
Expression profiles of DEGs involved in lignin biosynthesis within the phenylpropanoid pathway in wheat roots under zinc deficiency. Red and blue represent up- and downregulated genes, respectively. PAL: phenylalanine ammonia lyase; C4H: cinnamate 4-hydroxylase; C3H: p-coumarate 3-hydroxylase; COMT: caffeic acid O-methyltransferase; F5H: ferulate 5-hydroxylase; 4CL: 4-coumarate: CoA ligase; HCT: hydroxycinnamoyl transferase; CCoAOMT: caffeoyl-CoA O-methyltransferase; CCR: cinnamoyl-CoA reductase; CAD: cinnamyl alcohol dehydrogenase; LAC: laccases; POD: peroxidase; FC: fold change.

In this study, zinc deficiency markedly suppressed the expression of DEGs belonging to *CCR*, *CAD*, and *COMT* gene families (Figure 8). Specifically, seven *CCR*, two *CAD*, and one *COMT* genes exhibited significant downregulation, with fold change (FC) values ranging from 0.03 to 0.34. Additionally, although POD and LAC are crucial for the final polymerization step of lignin formation in plant cell walls, only *POD* genes showed upregulation (six DEGs, FC = 2.02 to 3.50), while three *LAC* genes (*TraesCS1B02G05690*, *TraesCS3B02G489700*, and *TraesCS3D02G444900*) were significantly downregulated (FC = 0.08 to 0.09). These findings suggest that zinc deficiency suppresses lignin biosynthesis by downregulating key genes involved in both monomer production and polymerization.

### 2.8. Upregulation of DEGs Involved in S-Adenosylmethionine and Ethylene Biosynthesis Under Zinc Deficiency

**Figure 9:**
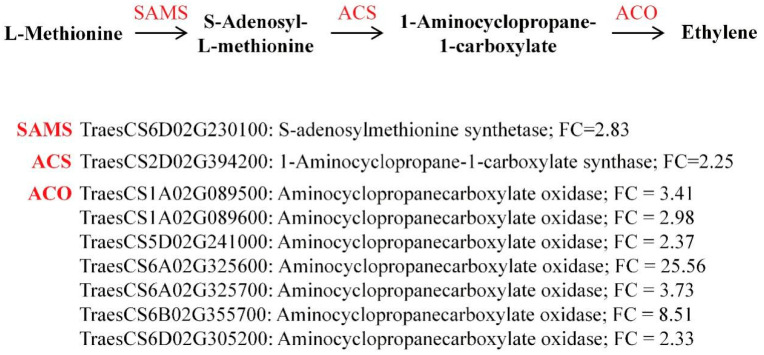
Expression profiles of DEGs involved in S-adenosylmethionine and ethylene biosynthesis in the cysteine and methionine metabolism pathway in the aboveground parts of wheat under zinc deficiency stress.

KEGG pathway analysis also identified “cysteine and methionine metabolism” as one of the most enriched pathways in the aboveground parts under zinc deficiency, with most DEGs showing significant upregulation (Figure 9). S-adenosylmethionine synthase (SAMS) catalyzes the production of S-adenosylmethionine (SAM), a precursor for both nicotianamine (NA) and ethylene (ET). NA, and its derivative mugineic acids (MAs), are critical metal chelators involved in zinc and iron transport, synthesized via the action of NAS, NAAT, and DMAS.

Under zinc-deficient conditions, the *SAMS* gene (*TraesCS6D02G230100*) was significantly upregulated in the aboveground tissues (FC = 2.83). In addition, four *NAS* genes and one *DMAS* gene in the roots, along with one NAAT gene in the shoots, were strongly induced (Table 3), indicating activation of chelator biosynthesis under zinc deficiency.

SAM also serves as the precursor for ethylene biosynthesis. It is converted to 1-aminocyclopropane-1-carboxylate (ACC) by ACC synthase (ACS)—the rate-limiting enzyme in ethylene production—and then to ethylene by ACC oxidase (ACO). Under zinc deficiency, expression of one *ACS* gene and seven *ACO* genes was significantly upregulated, with FC values ranging from 2.37 to 25.56. These data suggest that zinc deficiency enhances the biosynthesis of SAM, NA, MAs, and ethylene, possibly as part of an adaptive response.

### 2.9. qPCR Validation of RNA-Seq-Identified DEGs

**Figure 10:**
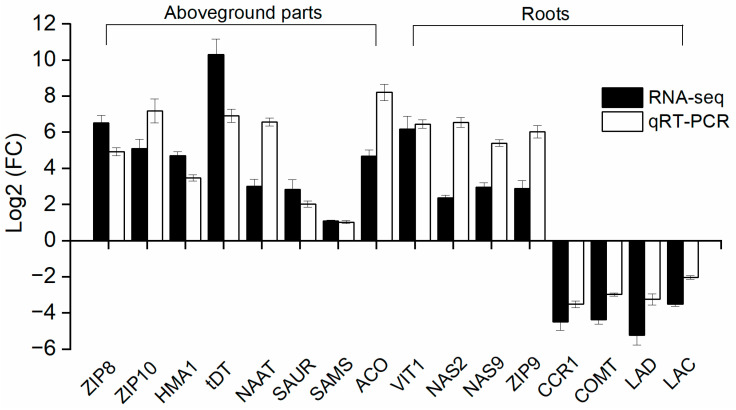
qPCR validation of 16 DEGs in the roots and aboveground parts of wheat seedlings under zinc deficiency. Error bars represent the SD of three biological replicates. FC: fold change. ZIP: ZRT- and IRT-like proteins; VIT: vacuolar iron transporter; HMA: heavy metal ATPase; NAS: nicotianamine synthase; SAUR: auxin response protein; CCR: cinnamoyl-CoA reductase; CAD: cinnamyl alcohol dehydrogenase; SAMS: S-adenosylmethionine synthase; ACO: ACC oxidase; NAAT: nicotianamine aminotransferase; tDT: tonoplast dicarboxylate transporter.

To validate the RNA-Seq results, 16 DEGs were selected based on their differential expression levels and biological relevance. These genes included those involved in zinc transport (*VIT1*, *ZIP8*, *ZIP9*, *ZIP10*, and *HMA1*), auxin response (*SAUR*), lignin biosynthesis (*CCR1*, *CAD*, and *LAC*), mugineic acid synthesis (*NAS8*, *NAS9*, *NAAT*, *DMAS*, and *SAMS*), ethylene biosynthesis (*ACO*), and organic acid transport (*tDT*). Quantitative PCR (qPCR) analysis confirmed that the expression patterns of all selected genes closely matched those observed in the RNA-Seq dataset, supporting the reliability and accuracy of the transcriptomic analysis (Figure 10).

## 3. Discussion

Zinc is an essential micronutrient for both plants and humans. In most plant species, tissue Zn concentrations typically range from 20 to 100 mg·kg^−1^, with levels below this range leading to symptoms of Zn deficiency [28]. Adequate Zn supply is critical for the proper functioning of metabolic processes, including carbohydrate and protein metabolism, auxin signaling, and overall reproductive development in plants and other organisms [12]. As primary producers in the food chain, plants play a vital role in human nutrition. Therefore, enhancing Zn uptake from the soil and improving its accumulation in edible parts such as wheat and rice holds substantial potential for addressing global Zn malnutrition [27].

The root system is the primary organ responsible for nutrient and water acquisition, directly influencing plant growth and development [29]. Zinc deficiency can significantly alter root morphology and activity, which, in turn, affects Zn uptake efficiency [30,31]. Previous studies have shown that species like barley (Hordeum vulgare), rice (Oryza sativa), and Malus hupehensis exhibit increased root length and surface area under short-term Zn deficiency [31,32,33]. Consistent with these findings, our study demonstrated that the Zn-efficient wheat cultivar “Zhongmai 175” developed significantly longer roots after two weeks of Zn deprivation, although increases in root surface area, volume, diameter, and tip number were not statistically significant. These results suggest that during early Zn deficiency, wheat may enhance Zn uptake by promoting root system activity. However, prolonged Zn deficiency can eventually suppress plant growth due to sustained depletion of internal Zn reserves [34,35,36].

Organic acids such as oxalic, citric, malic, acetic, and succinic acids are known to influence mineral uptake and transport in plants [25]. Their secretion into the rhizosphere under nutrient-deficient conditions lowers soil pH and mobilizes insoluble Zn, thus enhancing its availability to plants [37,38,39,40]. For instance, oxalic acid levels are significantly higher in high Pi-efficient wheat genotypes compared to their low-efficiency counterparts [41], and a positive correlation has been observed between soluble Zn and the concentrations of malic and oxalic acids in the aerial parts of *Thlaspi caerulescens* [42]. Zn is primarily transported in the xylem of soybean and tomato as Zn-citrate or Zn-malate complexes [26]. A similar mechanism occurs in rice, where Zn deficiency induces oxalic acid secretion, and citric acid exudation is correlated with Zn deficiency tolerance [43,44]. The tonoplast dicarboxylate transporter (tDT), which mediates malic acid transport across vacuolar membranes, plays a pivotal role in the transmembrane trafficking of Zn-malate complexes [45]. In our study, Zn deficiency significantly increased the secretion of four organic acids—oxalic, malic, acetic, and succinic acid—with oxalic and malic acids rising by 1.43-fold and 2.25-fold, respectively. Furthermore, several *tDT* genes were significantly upregulated in both roots and shoots under Zn-deficient conditions. These results, consistent with prior reports, underscore the integral roles of organic acids and *tDT* in maintaining Zn homeostasis in wheat under Zn-deficient stress.

Transporter proteins are fundamental for Zn uptake, translocation, distribution, and accumulation in plants [46]. A total of 58 ZIP genes have been identified in wheat through a genome-wide search against the IWGSC v1.1 reference genome, with six of them showing strong responses to Zn-Fe deficiency [47]. Notably, *TaZIP14-B* and *TaZIP13-B* were shown to transport both Zn and Fe in yeast complementation assays. In rice, vacuolar iron transporter genes OsVIT1 and OsVIT2 contribute to the sequestration of Fe, Zn, and Mn into vacuoles. Disruption of these genes leads to elevated Zn and Fe accumulation in seeds and reduced levels in flag leaves [48]. Similarly, *HMA4.1* and *HMA4.2* in tobacco are involved in root-to-shoot Zn translocation, and *HMA4* knockdown mutants exhibit markedly reduced foliar Zn levels [22]. Moreover, mugineic acids (MAs), synthesized through the methionine cycle, enhance Zn solubility and mobility in the rhizosphere, thereby promoting Zn accumulation in plant tissues [49,50]. Our transcriptomic data revealed that DEGs under Zn-deficient conditions were significantly enriched in biological processes related to ion transmembrane transport, Zn ion transport, and Zn homeostasis. These DEGs included genes from the *ZIP*, *VIT*, *HMA*, *NAS*, and *NAAT* families, all of which were upregulated in response to Zn deficiency. These findings highlight the coordinated involvement of key transporter families in Zn acquisition and distribution in wheat under Zn-limited conditions.

Cell wall remodeling also plays a significant role in plant responses to metal stress. It is well established that cell walls can bind and sequester toxic metal ions such as Cu (II), Zn (II), Fe (III), Cd (II), and Pb (II), thereby reducing their cytoplasmic entry and toxicity [51,52,53]. Lignin, a key component of the secondary cell wall synthesized via the phenylpropanoid pathway, contributes to this defensive mechanism [54]. It also plays a role in the formation of the Casparian strip, which regulates the selective uptake and exclusion of solutes in roots [55]. Under excess heavy metal stress, lignin accumulation increases, thickening the cell wall and limiting the translocation of toxic ions into root cells [56,57]. For example, in the Zn/Cd hyperaccumulator *T. caerulescens*, lignin biosynthetic genes such as *PAL*, *CAD*, and *CCR* are upregulated in response to high Zn exposure [58]. Similarly, Al (III) stress in rice enhances the expression of lignin-related genes, including *4CL*, *CAD*, *C3H*, and *PAL*, resulting in increased lignin deposition in endodermal cell walls and improved Zn/Cd sequestration [59].

Interestingly, our study found the opposite response under Zn-deficient conditions. Expression of lignin biosynthesis genes belonging to the *CCR*, *CAD*, *COMT*, and *LAC* families was significantly downregulated in wheat roots (Figure 8). *CCR*, *CAD*, and *COMT* encode core enzymes in the monolignol biosynthesis pathway, while *LAC* genes encode laccases responsible for the final oxidative polymerization step in lignin formation [60,61,62]. Dual silencing of *CCR* and *CAD* has been shown to markedly reduce lignin content in tobacco [63], and triple mutants of *LAC* genes (*lac4lac11lac17*) nearly abolish lignin deposition in *Arabidopsis* [64]. The coordinated downregulation of these genes under Zn-deficient conditions suggests that reduced lignin synthesis may enhance Zn mobility and uptake by decreasing Zn sequestration in the cell wall. This phenomenon was also reported in rice [65] and *Areca catechu* under similar Zn-deficient conditions [66].

Root exudates play a critical role in plant adaptation to Zn and Fe deficiency. Under Fe-deficient conditions, plants acquire Fe (III) through the secretion of phytosiderophores (PSs), which are low molecular weight metal-chelating compounds. Among these, NA and MAs exhibit a high affinity for Fe (III), forming soluble Fe–phytosiderophore complexes that are subsequently taken up by root cells via a high-affinity transport system. Recent studies have shown that, in addition to Fe (III), MAs and NA can also chelate Zn (II), thereby enhancing Zn availability in the rhizosphere and contributing to increased Zn accumulation in plants overexpressing NAS genes [67]. For example, barley secretes higher levels of MAs under Zn-deficient conditions, facilitating the formation of Zn (II)–MA complexes that promote Zn uptake by roots [24]. Likewise, significantly greater amounts of MAs have been detected in the rhizosphere of Zn-efficient wheat cultivars compared to Zn-inefficient ones [68].

MAs are synthesized from L-methionine via a conserved biosynthetic pathway involving several enzymes: SAMS, NAS, NAAT, and DMAS. NAS catalyzes the trimerization of SAM to produce NA. NA is then converted into a 3′-keto intermediate through the transfer of an amino group by NAAT, followed by reduction at the 3′-carbon to yield 2′- DMA, the first MA synthesized in this pathway [69,70]. In rice, genes such as *OsNAS*, *OsDMAS*, *OsSAMS*, and *OsNAAT* are involved in the biosynthesis, transport, and secretion of PSs in the root zone, thereby enhancing metal acquisition [71]. Notably, expression of *NAS2* in rice improves dietary Fe and Zn content in wheat [72], and ectopic expression of *NAS* in transgenic tobacco results in increased Fe and Zn accumulation [73]. In our study, we identified significant upregulation of one *NAS2*, three *NAS9*, and one *DMAS* gene in wheat roots, along with one *NAAT* gene in aboveground tissues, under Zn-deficiency stress. These findings confirm that genes and gene networks associated with PS biosynthesis are integrally involved in wheat responses to Zn deficiency and play a central role in Zn homeostasis.

Ethylene, a gaseous phytohormone, regulates diverse physiological processes including seed germination, root development, fruit ripening, and organ senescence. In the ethylene biosynthetic pathway, SAM is converted to ACC by ACS, which catalyzes the rate-limiting step. ACC is then converted to ethylene by ACO, members of a large family of oxygenases and oxidases [74]. Ethylene has also been implicated in plant responses to Fe deficiency. During early Fe deficiency, expression of SAMS, ACS, and ACO—particularly ACS—is upregulated, leading to a marked increase in ethylene production [75]. Concurrently, Fe deficiency induces changes in the expression of ethylene signaling genes such as ETR, EIN2, and EIN3 [76]. Furthermore, ethylene has been shown to positively regulate AtNAS and AtFRD3, two key genes involved in enhancing long-distance Fe transport [77].

In the present study, we observed that under Zn-deficient conditions, the expression of *SAMS*, *ACS*, and *ACO* in the cysteine and methionine metabolism pathway, as well as EIN3 and ETR in the plant hormone signal transduction pathway, was significantly upregulated in aboveground wheat tissues. These results suggest that, akin to Fe deficiency, ethylene signaling may play a regulatory role in plant adaptation to Zn deficiency. This is consistent with the biochemical and physiological similarities in the uptake and transport mechanisms of Fe and Zn [78].

## 4. Materials and Methods

### 4.1. Plant Materials and Growth Conditions

To evaluate zinc efficiency across 42 wheat varieties, a field experiment was conducted in 2024 at the Agricultural Experimental Farm of Anhui Science and Technology University. The physicochemical properties of the experimental soil are detailed in Table 4. Each wheat variety was sown in 10 rows, with 80 seeds per 4 m-long row and a row spacing of 0.25 m. Field management adhered to local standard agronomic practices. At maturity, 50 wheat spikes per variety were randomly collected via blind sampling [79], and air-dried in nylon bags, followed by manual threshing. Approximately 50 g of grains were air-dried in nylon bags, manually threshed, and approximately 50 g of grains per sample were rinsed three times each with tap and distilled water. The samples were dried at 65 °C to constant weight and ground into powder using a Laboratory 3100 cross-beater mill (Perten Instruments, Hamburg, Germany) for subsequent zinc content analysis.

For zinc deficiency treatments, hydroponic experiments were conducted using Zhongmai 175, a high zinc efficiency wheat cultivar. Plants were cultivated in modified Hoagland nutrient solution containing: 5 mM Ca (NO_3_)_2_, 5 mM KNO_3_, 1 mM KH_2_PO_4_, 2 mM MgSO_4_, 46 μM H_3_BO_3_, 10 μM MnSO_4_, 2 μM ZnSO_4_, 0.05 μM (NH_4_)_6_Mo_7_O_24_, 0.32 μM CuSO_4_, and 50 μM NaFeEDTA [23]. Plants were grown in controlled environment chambers (GXZ-300B; JNYQ, China) under a 14 h photoperiod with a photosynthetically active radiation (PAR) of 250 µmol m^−2^ s^−1^, at 25 °C during the day and 18 °C at night.

Plump seeds were surface-sterilized in 1% NaClO for 5 min, rinsed three times with sterile distilled water, and germinated on moist filter paper in the dark. After seven days, uniformly germinated seedlings were transferred to black plastic hydroponic boxes (122 mm × 82 mm × 113 mm), each containing 800 mL of Hoagland solution. Fourteen-day-old seedlings at the two-leaf stage were subjected to zinc treatments: control (CK, 2 μM Zn) and zinc-deficient (-Zn, 0 μM Zn) [23]. The nutrient solution was refreshed every three days. Thirty seedlings per box were pooled as a biological replicate, and each treatment included three biological replicates in a completely randomized design. After two weeks of treatment, root and leaf samples were collected for RNA sequencing, root morphology assessment, and root exudate analysis.

### 4.2. Root Morphological Scanning

Fresh roots were soaked in tap water for 10 min and then rinsed thoroughly. Roots were carefully spread to avoid overlap and scanned using the ScanMaker i800 Plus (Shanghai, China). Root morphological traits—including total root length, surface area, volume, average diameter, and number of root tips—were quantified using the LA-S Plant Root Analysis System (Hangzhou, China).

### 4.3. Determination of Organic Acids in Root Exudates

Root exudates were collected two weeks after zinc deficiency treatment, following a previously described method [80]. Plants were removed from the nutrient solution, and root systems were rinsed three times with deionized water. The roots were then incubated in a plastic tray containing 100 mL deionized water for 2 h. To prevent microbial degradation, thymol was immediately added to the collection solution. The exudate was filtered and concentrated to dryness at 55 °C using a temperature-controlled concentrator. The residue was reconstituted in 10 mL deionized water and stored at 0 °C in light-proof containers.

The root volume was measured using water displacement after each collection. All samples were filtered through a 0.45 µm membrane prior to high-performance liquid chromatography (HPLC) analysis (Agilent 1260 series). Chromatographic conditions were: Polaris 180A C18-A column (250 mm × 4.6 mm, 5 µm); mobile phase of 0.5% NH_4_H_2_PO_4_ aqueous solution at pH 2.8; flow rate of 1.0 mL/min; and injection volume of 10 µL. Identification of organic acids was based on retention time comparison with standard compounds, and quantification was performed using external calibration curves. Standard-grade oxalic acid, tartaric acid, malic acid, and citric acid were procured from Sigma-Aldrich (Darmstadt, Germany).

### 4.4. RNA-Sequencing

Total RNA was extracted from root and leaf samples of both control and zinc-deficient plants using an EasyPure™ Plant RNA Kit (Transgene Biotech, Beijing, China), following the manufacturer’s protocol. RNA concentration and quality were assessed using a Nanodrop 2000 spectrophotometer and Agilent 2100 Bioanalyzer with an RNA 6000 Nano Kit. mRNA was enriched using VAHTS™ mRNA Capture Beads (Vazyme, Nanjing, China) and fragmented using divalent cations at high temperature. First-strand cDNA was synthesized using random hexamer primers, followed by second-strand synthesis and purification with a QiaQuick PCR Extraction Kit (Qiagen, Shanghai, China). After end repair, A-tailing, and adapter ligation, the cDNA library was amplified and purified.

Library quality was initially assessed using Qubit 2.0, and insert sizes were verified using the Agilent 2100 Bioanalyzer. Quantitative PCR (qPCR) was then used to measure effective library concentration. High-throughput sequencing was performed using paired-end 150 bp reads on the Illumina HiSeq™ 2500 platform. All sequencing data have been submitted to the NCBI SRA database under accession number PRJNA1290848.

### 4.5. Differential Gene Expression Analysis

Raw sequencing reads were filtered to remove low-quality reads (defined as those with >20% of bases having quality scores <10), adapter sequences, and reads containing more than 5% ambiguous bases (N). Clean reads were aligned to the wheat reference genome IWGSC RefSeq v2.1 using the corresponding annotation v2.0 (https://urgi.versailles.inra.fr/download/iwgsc/IWGSC_RefSeq_Assemblies/v2.0/, accessed on 3 April 2025) [18]. SAM files were converted to BAM format using SAMtools, and HISAT2 v2.4.0 (http://daehwankimlab.github.io/hisat2/download/; accessed on 18 March 2023) was used for alignment. The mapped reads of each sample were assembled by StringTie (v1.3.3b) in a reference-based approach. For quantification of gene expression level features, featureCounts v.1.6.3 (https://doi.org/10.1093/bioinformatics/btt656, accessed on 5 April 2025) software was used to count the read numbers mapped to each gene.

Functional annotation of assembled transcripts was performed using BLASTX (E-value threshold = 1 × 10^−5^) against multiple databases: NCBI Nr, Nt, Swiss-Prot, PFAM, Gene Ontology (GO), EuKaryotic Orthologous Groups (KOG), and Kyoto Encyclopedia of Genes and Genomes (KEGG) [19]. Gene expression levels were normalized to FPKM (Fragments Per Kilobase of transcript per Million mapped reads). The Pearson correlation coefficients (PCCs) were calculated between the FPKM of three replicates of each sample using the built-in core function in the R software (www.r-project.org/) (V3.5.1).

Differentially expressed genes (DEGs) were identified using the DESeq2 R package (v1.40.2). Genes with a false discovery rate (FDR)-adjusted *p*-value < 0.05 and absolute log_2_ fold change ≥ 1 were considered significant DEGs. GO and KEGG enrichment analyses were conducted using topGO (v2.50.0) and KOBAS (v3.0), respectively, with significance defined as adjusted *p* ≤ 0.05. Visualization of DEGs and enriched pathways was performed using the pheatmap R package (v1.0.12) in RStudio (v2025.05.0).

### 4.6. qRT-PCR Analysis

Quantitative real-time PCR (qRT-PCR) was performed using TB Green^®^ Premix Ex Taq™ II (Takara, Beijing, China) according to the manufacturer’s protocol on a ViiA 7 Real-Time PCR System (Thermo Fisher Scientific, Shanghai, China). Each reaction contained 1 µL of cDNA (derived from either CK or -Zn treatments), 1 µL each of forward and reverse primers (10 µM), 10 µL of TB Green Fast qPCR Mix, and 7 µL of RNase-free water. Primers were designed using Primer 5 software and are listed in Supplementary Table S1. *TaActin* (TraesCS1A02G274400) served as the internal reference gene. All reactions were performed in three independent technical replicates. Relative mRNA expression levels were calculated using the 2^−ΔΔCt^ method [81].

### 4.7. Statistical Analysis

Statistical analyses were conducted using IBM SPSS Statistics 29.0. Each experiment was repeated at least three times. One-way ANOVA followed by Duncan’s multiple range test was used to assess significant differences between the CK and -Zn treatments. Differences were considered statistically significant at *p* ≤ 0.05.

## 5. Conclusions

In this study, a high zinc deficiency-tolerant wheat cultivar was initially identified through field trials. Subsequent hydroponic experiments revealed that zinc deficiency stimulated the secretion of organic acids in root exudates and enhanced total root length at early developmental stages. To further elucidate the underlying molecular responses, transcriptome analysis was conducted. Gene Ontology enrichment indicated that DEGs were primarily involved in zinc ion transport, zinc ion homeostasis, ion transmembrane transport, and hormone-mediated signaling pathways. KEGG analysis highlighted phenylpropanoid biosynthesis and cysteine and methionine metabolism as the most significantly enriched pathways. These findings offer valuable insights into the molecular mechanisms by which wheat responds to zinc deficiency and lay a foundation for improving nutrient stress tolerance through molecular breeding.

## Figures and Tables

**Table 1:** Root morphological traits of Zhongmai 175 under Zn-deficient conditions.

Treatment	Total Root Length (cm)	Total Root Surface Area (cm^2^)	Total Root Volume (cm^3^)	Average Root Diameter (mm)	Number of Root Tips
CK	418.32 b	1.89 a	72.32 a	0.56 a	724 a
-Zn	489.03 a	2.08 a	81.19 a	0.63 a	827 a

CK: 2 µM; -Zn: 0 µM. Different letters indicate significant differences between CK and -Zn groups (*p* < 0.05). Values are means of three replicates.

**Table 2:** Summary statistics of RNA-seq data.

Sample	Raw Reads	Clean Reads	Mapped Reads	Mapped (%)	Unique Mapped Reads (%)	Clean Q30 (%)	Exon (%)
CKR1	76,657,956	75,174,400	64,536,355	85.85	79.40	95.40	82.78
CKR2	73,554,322	72,111,182	65,898,408	91.38	76.76	95.58	83.30
CKR3	81,630,228	80,045,472	68,349,124	85.39	79.11	95.52	83.33
-ZnR1	72,715,286	71,350,538	65,855,677	92.30	72.40	95.58	80.86
-ZnR2	79,714,610	78,245,238	70,729,786	90.40	83.33	95.34	82.26
-ZnR3	79,028,732	77,640,002	68,806,972	88.62	82.04	95.59	81.92
CKA1	88,092,076	86,186,994	79,824,523	92.62	85.39	95.22	82.73
CKA2	84,953,578	83,296,162	78,667,403	94.44	86.32	95.47	82.75
CKA3	81,094,022	79,433,574	75,224,060	97.70	87.86	95.38	82.29
-ZnA1	76,738,728	75,184,032	71,478,194	95.07	88.19	95.41	83.48
-ZnA2	83,440,878	81,729,870	76,978,018	94.19	86.68	95.29	83.20
-ZnA3	76,232,316	74,721,754	70,389,855	94.20	86.84	95.54	80.82

CKR: roots under Zn-sufficient conditions; -ZnR: roots under Zn-deficient conditions; CKA: aboveground parts under Zn-sufficient conditions; -ZnA: aboveground parts under Zn-deficient conditions.

**Table 3:** DEGs involved in zinc ion transport.

Gene ID (-ZnR_vs._CKR_up)	Log_2_ (FC)	*p* Value	Gene Description
*TraesCS7D02G413000*	6.17	1.39 × 10^−32^	vacuolar iron transporter 1 (VIT1-7D)
*TraesCS5B02G202100*	1.15	5.88 × 10^−5^	vacuolar iron transporter 2 (VIT2-5B)
*TraesCS5A02G552400*	2.38	1.70 × 10^−6^	nicotianamine synthase 2 (NAS2-5A)
*TraesCS2D02G094200*	2.97	1.86 × 10^−16^	nicotianamine synthase 9 (NAS9-2D)
*TraesCS2B02G111100*	2.97	8.01 × 10^−8^	nicotianamine synthase 9 (NAS9-2B)
*TraesCS2A02G095700*	1.75	3.06 × 10^−5^	nicotianamine synthase 9 (NAS9-2A)
*TraesCS2B02G023500*	2.59	3.41 × 10^−5^	deoxymugineic acid synthase (DMAS-2B)
*TraesCS1D02G294000*	2.89	5.75 × 10^−9^	ZRT- and IRT-like proteins 9 (ZIP9-1D)
*TraesCS4A02G025400*	1.86	1.29 × 10^−7^	ZRT- and IRT-like proteins 9 (ZIP9-4A)
*TraesCS4D02G277100*	1.81	7.62 × 10^−5^	ZRT- and IRT-like proteins 9 (ZIP9-4D)
*TraesCS7B02G321200*	1.80	5.46 × 10^−5^	ZRT- and IRT-like proteins 10 (ZIP10-7B)
*TraesCS7A02G420600*	2.41	1.03 × 10^−7^	ZRT- and IRT-like proteins 10 (ZIP10-7A)
*TraesCS7D02G412800*	1.43	3.3 × 10^−6^	cadmium/zinc-transporting ATPase (HMA2-7D)
*TraesCS5B02G261800*	2.40	8.80 × 10^−5^	tonoplast dicarboxylate transporter (tDT-5B)
*TraesCS5B02G261500*	2.01	3.42 × 10^−7^	tonoplast dicarboxylate transporter (tDT-5B)
*TraesCS3A02G183800*	1.75	4.42 × 10^−5^	tonoplast dicarboxylate transporter (tDT-3A)
*TraesCS3D02G188000*	2.42	1.98 × 10^−7^	tonoplast dicarboxylate transporter (tDT-3B)
Gene ID (-ZnA_vs._CKA_up)	Log_2_ (FC)	*p* Value	Gene Description
*TraesCS2A02G143400*	6.52	1.87 × 10^−10^	ZRT- and IRT-like proteins 8 (ZIP8-2A)
*TraesCS2D02G146800*	4.84	5.03 × 10^−5^	ZRT and IRT-like proteins 8 (ZIP8-2D)
*TraesCS1A02G297400*	2.91	6.55 × 10^−6^	ZRT- and IRT-like proteins 9 (ZIP9-1A)
*TraesCS1B02G306400*	3.85	8.74 × 10^−6^	ZRT- and IRT-like proteins 9 (ZIP9-1B)
*TraesCS7D02G413300*	5.11	1.33 × 10^−8^	ZRT- and IRT-like proteins 10 (ZnT10-7D)
*TraesCS5A02G230300*	4.71	1.68 × 10^−6^	cadmium/zinc-transporting ATPase (HMA1-5A)
*TraesCS7D02G412800*	2.67	2.12 × 10^−6^	cadmium/zinc-transporting ATPase (HMA2-7D)
*TraesCS7B02G448000*	4.95	6.64 × 10^−5^	basic leucine zipper 19 (bZIP19-7B)
*TraesCS3D02G188000*	10.32	1.78 × 10^−13^	tonoplast dicarboxylate transporter (Tdt-3D)
*TraesCS3A02G183800*	6.97	4.18 × 10^−6^	tonoplast dicarboxylate transporter (tDT-3A)
*TraesCS5A02G263100*	5.99	1.45 × 10^−6^	tonoplast dicarboxylate transporter (tDT-5A)
*TraesCS5D02G270600*	2.89	4.52 × 10^−5^	tonoplast dicarboxylate transporter (tDT-5D)
*TraesCS3A02G183800*	6.97	4.18 × 10^−6^	tonoplast dicarboxylate transporter (tDT-3A)
*TraesCS1B02G300600*	3.02	4.78 × 10^−5^	nicotianamine aminotransferase A (NAAT-1B)

**Table 4:** Physicochemical properties of the experimental soil.

Organic Matter (g·kg^−1^)	Total Nitrogen (g·kg^−1^)	Available Phosphorus (mg·kg^−1^)	Available Potassium (mg·kg^−1^)	Available Zinc (mg·kg^−1^)	pH
21.36	1.16	22.10	150.56	0.85	6.89

## Data Availability

All raw sequencing data are available in the NCBI Sequence Read Archive under accession number PRJNA1290848.

## Supplementary Materials

The following supporting information can be downloaded at: https://www.mdpi.com/article/10.3390/biology14080985/s1, Table S1. List of primers used in the study; Table S2. Differentially expressed gene classification in roots; Table S3. Differentially expressed gene classification in aboveground parts; Table S4. GO enrichment analysis of DEGs in roots; Table S5. GO enrichment analysis of DEGs in aboveground parts; Table S6. KEGG enrichment analysis of DEGs in roots; Table S7. KEGG enrichment analysis of DEGs in the aboveground parts.

## Abbreviations

Zn	Zinc
Fe	Iron
ZIP	ZRT, IRT-like protein
DMA	Deoxymugineic acid
HMA	Heavy metal ATPase
MAs	Mugineic acids
VIT	Vacuolar iron transporter
NAS	Nicotianamine synthase
DMAS	Deoxymugineic acid synthase
tDT	Tonoplast dicarboxylate transporter
NAAT	Nicotianamine aminotransferase
FC	Fold change
CCR	Cinnamoyl-CoA reductase
CAD	Cinnamyl alcohol dehydrogenase
COMT	Caffeic acid-O-methyltransferase
LAC	Laccases
EIN3	Ethylene-insensitive protein 3
ETR	Ethylene receptor
ACO	1-aminocyclopropane-1-carboxylate oxidase
ACS	1-aminocyclopropane-1-carboxylate
SAMS	S-adenosylmethionine synthase
NA	Nicotianamine
PSs	Phytosiderophores
