# Supplementary material for: Transcriptome Analysis Reveals Candidate Pathways and Genes Involved in Wheat (Triticum aestivum L.) Response to Zinc Deficiency
Source: Biology (Basel). 2025 Aug 2;14(8):985. doi: 10.3390/biology14080985 (PMC12383756; doi:10.3390/biology14080985)
Supplement: Supplementary file 1 [file biology-14-00985-s001.zip › Table S1. List of primers used in the study.pdf]

Table S1. List of primers used in the study.

No.	Gene ID	Description and gene name	Primer (5'-3')
1	TraesCS7D02G413000	Vacuolar iron transporter, VIT1	F: ACAGTGTTGGTGAGGGACGG<br>R: GAGGACGAGCGAGGATGGA
2	TraesCS2A02G143400	ZRT- and IRT-like proteins, ZIP8	F: GCGGGGCTATGGGTTGCT<br>R: CGTCGGGGAGGATGTGGAT
3	TraesCS1D02G294000	ZRT- and IRT-like proteins, ZIP9	F: CCTCTTCTTCGGCGTCAAGG<br>R: CGTGCTGGTGCTCTGGGAT
4	TraesCS7A02G420600	ZRT- and IRT-like proteins, ZIP10	F: TCGCCGCTATCCTTGTCTCG<br>R: CCTGTAGTCCTCCCGTCCTCC
5	TraesCS5A02G230300	Heavy metal ATPase, HMA1	F: CCCCACCTCCCATCCAAG<br>R: AGAGTGCATTACCCCGACAAGA
6	TraesCS5A02G552400	Nicotianamine synthase, NAS2	F: TGAAATGGCTGCCCAGAACA<br>R: TCGGAGCAGAGGCGGATG
7	TraesCS2D02G094200	Nicotianamine synthase, NAS9	F: GAGGGGTGCTGCGACAAGA<br>R: GAAGTAGGGGAAGAGGGTGAGG
8	TraesCS5B02G212600	Auxin response protein, SAUR	F: GCACCAAGCTCGCCAGAGT<br>R: AATAGCCCCAAAACAGCATCAT
9	TraesCS6B02G420000	Cinnamoyl-CoA reductase, CCR1	F: AAGCCCACCTACCCGAATGC<br>R: GCGGTTATGTTGTACTGAGGAAAGA
10	TraesCS5A02G205700	Cinnamyl alcohol dehydrogenase, CAD	F: AGGGTTCAGAAGGACGAGGATG<br>R: GGAACGACGGGGTAGAGGG
11	TraesCS1B02G056900	Laccases, LAC	F: GCCCACTTCTCCTTCCTCCG<br>R: TTTGCGTTCCACCACTCCC
12	TraesCS6D02G230100	S-adenosylmethione, SAMS	F: CTCATCTCCACCCAGCACGAC<br>R: ACAGACAAAGGCTCAGGCACA
13	TraesCS6A02G325600	1-aminocyclopropane-1-carboxylate oxidase, ACO	F: ACCAACGGGCGGTACAAGA<br>R: GGGCAGGGAAGATGACGG
14	TraesCS1B02G300600	Nicotianamine aminotransferase A, NAAT	F: TGGCAGAGCACTTGTCGCA<br>R: CCTGGGGAGCAGAATGTTGG
15	TraesCS3D02G188000	Tonoplast dicarboxylate transporter, tDT	F: TCCTGGTGGGGATGTGGTC<br>R: TCCTGAGATGGGTCCTGTCG
17	TraesCS1A02G274400	TaActin	F: TGGAAACGGCTAGGAGCAGC<br>R: ACCTCAGGGCACCTGAACCT
